# The effect of specific and rotating health warnings on smoking risk perception and quitting intentions: Evidence from China

**DOI:** 10.18332/tid/200106

**Published:** 2025-02-07

**Authors:** Kecheng Du, Gang Wang

**Affiliations:** 1 School of Journalism and Communication, Wuhan University, Wuhan, China; 2 Institute of Modern Communication Studies, School of Humanities and Communication, Ningbo University, Ningbo, China

**Keywords:** cigarette package warning labels, rotating health warnings, health risk perceptions, smoking cessation, tobacco control policy

## Abstract

**INTRODUCTION:**

This study aims to investigate whether non-rotating, specific health risk messages on cigarette packaging could be a practical alternative to rotating health warnings to improve smokers' health risk perceptions and intentions to quit smoking.

**METHODS:**

The study employs a cross-sectional randomized survey experiment conducted among 1700 adult smokers (aged ≥18 years) in China, with data collected using a snowball sampling method. Participants were randomly assigned to one of three groups: the control group (viewed standard packaging with a general health warning), the rotating health risk text group (exposed to four rotating disease-related warnings), and the reproductive health risk text group (focused on smoking’s impact on sexual health). After viewing the corresponding health warnings, participants reported their health risk perceptions and intentions to quit smoking, and responses to additional control variables.

**RESULTS:**

Rotating health risk text warnings on cigarette packaging significantly increased participants' perceptions of cardiovascular (β=0.20; 95% CI: 0.05–0.35), digestive (β=0.22; 95% CI: 0.07–0.37), respiratory (β=0.17; 95% CI: 0.07–0.26), and reproductive system risks (β=0.21; 95% CI: 0.06–0.37), while the non-rotating reproductive health risk text warnings only significantly improved perceptions of reproductive system risks (β=0.18; 95% CI: 0.10–0.25). Both types of text warnings significantly increased smokers’ intentions to quit smoking (p≤0.001), indicating that non-rotating specific health risk warnings can be equally effective in promoting quitting intentions.

**CONCLUSIONS:**

This study demonstrates that rotating health risk text on cigarette packaging offers comprehensive advantages in enhancing health risk perceptions. However, its effects on intentions to quit smoking are similar to those of non-rotating reproductive health risk warnings. These findings suggest that in contexts where implementing rotating warnings is challenging, non-rotating, specific health risk messages can serve as a feasible alternative to support the effective implementation of tobacco health warning policies.

## INTRODUCTION

The World Health Organization’s Framework Convention on Tobacco Control (FCTC) recommends that countries adopt rotating health warnings on tobacco products packaging and labelling to increase smokers’ awareness of health risks^1^. However, the implementation of this measure faces numerous challenges in practice, especially as it is unrealistic for every country to fully implement the necessary measures to successfully enforce rotating health warnings^2^. Additionally, commonly used rotating pictorial health warnings may bring about negative effects. Excessive health risk information may increase consumers’ cognitive load, potentially triggering psychological reactance, which could undermine the intervention effectiveness of health warnings^3,4^. In contrast, disseminating information about specific diseases with lower levels of public awareness may be more effective, significantly improving smokers’ cessation intentions and health risk awareness^5^.

In this context, this study uses a sample of Chinese smokers as an important case to explore whether providing specific disease risk information without adopting rotating health warnings can enhance the effectiveness of cigarette health warnings. Additionally, the study discusses the necessity of implementing rotating health warnings in China. Currently, China only requires text-based warning labels on cigarette packaging^6^. Although new cigarette warning label regulations were implemented in China in 2008, increasing the proportion of warning information on cigarette packaging; both old and new text warnings are non-rotating and do not specifically mention the diseases caused by smoking^7^. Many studies have shown that vague and generic text-based health warning labels have very limited warning effectiveness^8-13^. However, China’s unique political and economic context poses significant economic and political resistance to promoting more effective health warning measures^14^. The tobacco industry in China is operated by the state-owned China National Tobacco Corporation (CNTC), which is under the State Tobacco Monopoly Administration (STMA). As a state-owned enterprise, it contributes significantly to national fiscal revenue^15,16^. Therefore, implementing rotating pictorial health warnings on cigarette packaging, as required by the FCTC framework, may have a negative impact on tobacco sales^17^, further complicating the implementation process. Given the practical challenges of implementing pictorial warnings, this study focuses on the intervention effects of text-based rotating health warning information and non-rotating specific health warnings in improving Chinese smokers’ health risk awareness and cessation intentions.

This study hypothesizes that non-rotating specific health warning information can significantly improve Chinese smokers’ health risk awareness and cessation intentions, particularly when disseminating disease risk information with low public awareness. Research by Yang et al.^[Bibr CIT0018]18^ based on the International Tobacco Control China Wave 1 Survey, found that awareness of health risks related to sexual function is the weakest among all diseases, with only about 19.2% of respondents aware of the harms of smoking on sexual function. This awareness is much lower than that for lung cancer (approximately 73%) and coronary heart disease (approximately 40.2%). Therefore, this study selected sexual function disease risk as the content for the non-rotating specific health warning. Existing research suggests that this low-awareness risk information may have a greater marginal effect when conveyed through cigarette packaging health warnings, with a strong intervention effect, particularly among younger populations^5^. This study extends the conclusions of Zhang et al.^5^ by examining whether this intervention effect remains significant in a broader adult smoker sample. Through this investigation, this study aims to provide empirical evidence for the development of more feasible tobacco policies worldwide and offer theoretical and empirical support for optimizing cigarette health warning policies in China.

## METHODS

### Research design

**Figure 1:**
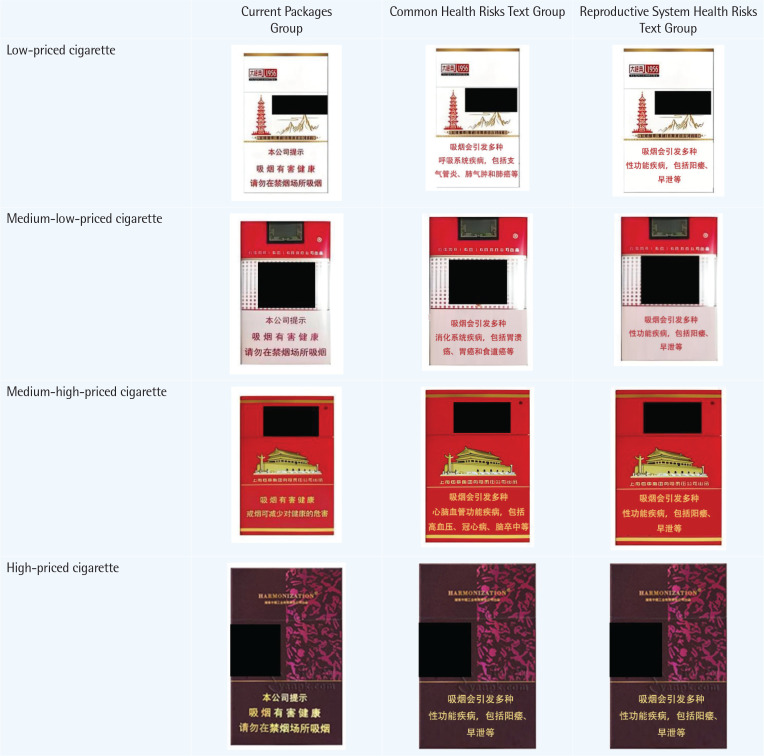
Warning packages and prices applied in survey experiment

This study adopts a survey experiment, combining the strengths of surveys and experiments to achieve a larger sample size^19^. The experiment selects four common types of cigarettes in the Chinese market – high-priced, medium-high-priced, medium-low-priced, and low-priced – as research objects (Figure 1). To examine the effects of rotating multiple health warning messages and using warnings focused on a specific disease, participants were divided into three groups, each exposed to different cigarette packaging images.

In the control group (Group 1), participants view commonly available cigarette packaging from the market, with a standard warning label stating: ‘Our company reminds you: Smoking is harmful to your health. Please refrain from smoking in public places’.

In the rotating health risk text group (Group 2), the text on the cigarette packaging is replaced with detailed descriptions of four common smoking-related diseases, including respiratory diseases, digestive diseases, cardiovascular diseases, and sexual dysfunction. Each warning is paired with a cigarette package from a different price tier, ensuring that participants in this group are exposed to all four disease warnings. The warnings include: ‘Smoking can lead to various respiratory diseases, including bronchitis, emphysema, and lung cancer’, ‘Smoking can cause numerous digestive diseases, including gastric ulcers, stomach cancer, and esophageal cancer’, ‘Smoking can result in cardiovascular diseases, including hypertension, coronary heart disease, and stroke’, and ‘Smoking increases the risk of sexual dysfunction, including impotence and premature ejaculation’. In practice, there are generally two specific methods for implementing rotation. One method is to simultaneously present various different health warning labels, so that smokers will randomly see different health warning labels when purchasing cigarettes. The other method is to set a time frame and periodically replace the health warning labels on cigarette packaging, so that smokers who buy cigarettes over a long period will randomly encounter different health warning labels. Since both methods aim to expose smokers to a greater variety of health warning labels, for practical purposes, this study simplifies the presentation of rotating information by displaying several different health warning labels simultaneously in the questionnaire.

In the reproductive health risk text group (Group 3), the warning on the packaging states: ‘Smoking can cause various sexual dysfunctions, including impotence and premature ejaculation’. This focus on reproductive health risks was chosen because most smokers are unaware of smoking’s effects on reproductive health^18^, potentially resulting in a higher marginal impact on their risk perception^5^.

### Participants and data collection

The target population for this study is Chinese adult smokers (aged ≥18 years). Participants were recruited using a snowball sampling method. In this study, 60 field investigators were randomly selected from undergraduate volunteers at universities in Wuhan, China, using a random number generator based on their student IDs.

In May 2024, we initially recruited 80 undergraduate students from a large research university. These students leverage their social networks to recruit participants through snowball sampling. Prior to data collection, the students were randomly assigned to one of three groups and tasked with distributing three different versions of the questionnaire within their networks. The control group was assigned 26 investigators, while the other two groups each had 27 investigators. The data collection spanned one month, resulting in a total sample size of 1700 participants: the control group included 564 participants exposed to existing cigarette packaging warnings; the rotating health risk text group comprised 559 participants exposed to detailed warnings about four smoking-related diseases paired with different price tiers; and the reproductive health risk text group consisted of 577 participants exposed to warnings focusing on reproductive health risks.

### Measures

The dependent variables in this study are participants’ perceptions of smoking-related health risks and their intentions to quit smoking. Following the completion of demographic questions, participants in the three groups were presented with images of the corresponding cigarette packaging (attached to the questionnaire) and subsequently asked to evaluate their perceptions of smoking risks and their intentions to quit within the context of the experimental conditions.

To assess overall perceptions of smoking risks, participants respond to the question: ‘To what extent do you agree that the cigarette packaging warning makes you think about the severity of smoking-related risks?’. Responses were recorded on a five-point Likert scale ranging from 1=strongly disagree to 5=strongly agree. Participants’ intentions to quit smoking were measured using the question: ‘To what extent do you agree that the cigarette packaging warning makes you think about quitting smoking?’. This item was also rated on a five-point Likert scale.

In addition, the study evaluated participants’ perceptions of health risks associated with specific physiological systems, including respiratory, digestive, cardiovascular, and reproductive systems. The corresponding questions were: ‘To what extent do you think tobacco harms your respiratory system?’, ‘To what extent do you think tobacco harms your digestive system?’, ‘To what extent do you think tobacco harms your cardiovascular system?’ and ‘To what extent do you think tobacco harms your sexual function?’. These items were measured using a four-point Likert scale, where 1=very little and 4=very much. These measures were adapted from previously validated survey instruments used in similar studies.

The survey also collected detailed demographic information, including age, gender, education level, income, marital status, and parental status. Age was calculated from participants’ reported year of birth. Gender was recorded as a binary variable (1=Male, 0=Female). Education level was measured on a seven-point scale, ranging from 1=Primary school to 7=Doctoral degree. Income was reported in units of 10000 RMB (1 RMB about US$0.15) through an open-ended question. Marital status was coded as a binary variable (0= single, 1=married/cohabitating/divorced/widowed), while parental status was similarly coded (0=No children, 1=Has children).

Nicotine dependence was included as a control variable in the statistical analysis and assessed through participants’ average daily cigarette consumption and the time elapsed before their first cigarette after waking. Time to the first cigarette was measured using a four-point scale: 1=within 5 minutes, 2=6–30 minutes, 3=31–60 minutes, and 4=after 61 minutes. Average daily cigarette consumption was also measured on a four-point scale: 1=10 cigarettes or fewer, 2=11–20 cigarettes, 3=21–30 cigarettes, and 4=31 cigarettes or more. These measures ensure robust control for nicotine dependence in subsequent analyses.

### Statistical analysis

All statistical analyses were conducted using Stata 15.1^20^. Descriptive statistics include means and standard deviations, providing an overview of the data distribution. Multivariable relationships were estimated using multiple linear regression models, with 95% confidence intervals reported to assess the precision of the estimates. All models account for demographic characteristics and nicotine dependence as control variables. Statistical significance was evaluated at three levels: p≤0.001, p≤0.01, and p≤0.05, with smaller p-values indicating stronger evidence to reject the null hypothesis in favor of the alternative hypothesis^21^. Adjusted R^2^ was used to evaluate the fit of the regression models. Unlike R^2^, which can increase simply by adding more predictors, adjusted R^2^ provides a more accurate measure by penalizing the inclusion of non-significant variables. Adjusted R^2^ is particularly meaningful for our regression analysis, as it accounts for the number of predictors and ensures a more reliable evaluation of model performance.

## RESULTS

### Descriptive analysis

Table 1 gives the means of the dependent variables across subgroups. Respondents’ perception of cardiovascular system risks had a mean score of 3.46 (SD=1.31), while their perception of digestive system risks averaged 3.49 (SD=1.29). Perceptions of respiratory system risks are higher, with a mean of 4.26 (SD=0.80), compared to reproductive system risks, which averaged 3.42 (SD=1.27). The overall perception of smoking-related health risks was 3.70 (SD=1.26), and the mean intention to quit smoking was 3.83 (SD=1.17). Variations across subgroups appear relatively minor.

**Table 1:** Descriptive statistics of survey participants, a survey experiment of the effect of specific and rotating health warnings on smoking risk perception and quitting intentions, China, 2024 (N=1700)

*Variables*	*Group 1* *Mean (SD)*	*Group 2* *Mean (SD)*	*Group 3* *Mean (SD)*	*All* *Mean (SD)*
Total	564	559	577	1700
Harmful to the cardiovascular system	3.39 (1.29)	3.61 (1.25)	3.37 (1.38)	3.46 (1.31)
Harmful to the digestive system	3.37 (1.27)	3.62 (1.31)	3.47 (1.28)	3.49 (1.29)
Harmful to the respiratory system	4.21 (0.89)	4.36 (0.74)	4.23 (0.75)	4.26 (0.80)
Harmful to the reproductive system	3.22 (1.36)	3.44 (1.30)	3.60 (1.12)	3.42 (1.27)
Perceived harm of personal smoking	3.55 (1.31)	3.82 (1.24)	3.72 (1.22)	3.70 (1.26)
Willingness to quit smoking	3.48 (1.33)	4.03 (1.11)	3.98 (0.98)	3.83 (1.17)
Age (years)	37.63 (12.24)	36.90 (12.64)	36.88 (12.29)	37.14 (12.39)
Gender	0.93 (0.26)	0.96 (0.21)	0.95 (0.21)	0.95 (0.23)
Education level	4.27 (1.13)	4.36 (1.15)	4.19 (1.23)	4.27 (1.17 )
Income	11.09 (6.61)	12.23 (9.03)	11.11 (7.12)	11.47 (7.66)
Marital status	0.78 (0.41)	0.72 (0.45)	0.78 (0.42)	0.76 (0.43)
Parental status	0.76 (0.43)	0.67 (0.47)	0.71 (0.46)	0.71 (0.45)
Cigarettes per day	2.21 (1.04)	2.33 (1.07)	2.35 (1.18)	2.30 (1.10)
Average waiting time from wakening to smoking the first cigarette	2.3 (1.14)	2.11 (1.08)	2.30 (1.10)	2.25 (1.11)

Among the participants, 95% were male. The average education level ranged between an associate degree and a Bachelor’s degree. The mean annual income was 114700 RMB (SD=128930). Regarding marital status, 76% of respondents were not single, and 71% had children. The average daily cigarette consumption of respondents ranged 11–20 cigarettes and 21–30 cigarettes per day, with a closer alignment to the 11–20 cigarettes per day category. And the typical time to the first cigarette after waking ranged between 6–30 minutes and 31–60 minutes, with a closer alignment to the 6–30 minutes category.

Tables 2–5 present the estimated results of the multivariable regression analyses. In these analyses, cases with missing responses for certain questions were automatically excluded from the sample. Models 1 in Tables 2 and 4, as well as Model 3 in Table 5, are based on the subsample comprising the control group (Group 1) and the rotating health risk text group (Group 2). After excluding cases with missing values, the total sample size was 1121. Models 2 in Tables 3 and 4, along with Model 4 in Table 5, are based on the subsample comprising the control group (Group 1) and the reproductive health risk text group (Group 3). After excluding cases with missing values, the total sample size was 1138.

**Table 2:** The impact of rotating health warnings on specific smoking risk perception, a survey experiment on smoking risk perception and quitting intentions, China, 2024 (Group 1 vs Group 2) (N=1121)

	*Cardiovascular* *system*		*Digestive system*		*Respiratory* *system*		*Reproductive* *system*	
	*Coefficient* *(95% CI)*	*p*	*Coefficient* *(95% CI)*	*p*	*Coefficient* *(95% CI)*	*p*	*Coefficient* *(95% CI)*	*p*
**Rotating health warnings**	0.20** (0.05–0.35)	0.01	0.22** (0.07–0.37)	0.01	0.17*** (0.07–0.26)	<0.001	0.21** (0.06–0.37)	0.01
**Control variables**								
Age (years)	-0.01 (-0.01–0.01)	0.12	-0.01 (-0.01–0.00)	0.09	-0.00 (-0.01–0.00)	0.53	0.00 (-0.01–0.01)	0.97
Gender	-0.02 (-0.33–0.30)	0.92	-0.03 (-0.36–0.29)	0.84	0.04 -0.17–0.25)	0.73	0.15 (-0.19–0.49)	0.39
Education level	0.20*** (0.14–0.27)	<0.001	0.06 (-0.01–0.13)	0.08	0.02 (-0.02–0.07)	0.30	0.03 (-0.04–0.11)	0.39
Income	0.01* (0.00–0.02)	0.05	0.01 (0.00–0.02)	0.09	0.00 (-0.00–0.01)	0.40	0.00 (-0.01–0.01)	0.40
Marital status	0.16 (-0.08–0.41)	0.19	0.10 (-0.15–0.36)	0.42	-0.01 (-0.17–0.15)	0.89	0.08 (-0.19–0.34)	0.56
Children	-0.12 (-0.35–0.12)	0.34	-0.19 (-0.44 – -0.05)	0.12	0.19* (0.04–0.35)	0.02	-0.20 (-0.45–0.05)	0.12
Cigarettes per day	-0.09 (-0.19–0.00)	0.06	-0.10* (-0.19–0.00)	0.05	0.07* (0.00–0.13)	0.03	0.09 (-0.01–0.19)	0.07
Average waiting time from wakening to smoking the first cigarette	-0.02 (-0.09–0.05)	0.60	-0.02 (-0.09–0.05)	0.57	-0.01 (-0.06–0.03)	0.59	-0.06 (-0.14–0.01)	0.09
Adj R^2^	0.05		0.02		0.01		0.01	

Multiple regression models: all models account for demographic characteristics and nicotine dependence as control variables.

*** p≤0.001,

** p≤0.01,

* p≤0.05.

**Table 3:** The impact of reproductive health risk warning on specific smoking risk perception, a survey experiment on smoking risk perception and quitting intentions, China, 2024 (Group 1 vs Group 3) (N=1138)

	*Cardiovascular* *system*		*Digestive system*		*Respiratory* *system*		*Reproductive* *system*	
	*Coefficient* *(95% CI)*	*p*	*Coefficient* *(95% CI)*	*p*	*Coefficient* *(95% CI)*	*p*	*Coefficient* *(95% CI)*	*p*
**Specific messages**	0.02 (-0.06–0.09)	0.67	0.05 (-0.03–0.12)	0.22	0.01 (-0.04–0.06)	0.59	0.18*** (0.10–0.25)	<0.001
**Control variables**								
Age (years)	-0.00 (-0.01–0.00)	0.23	0.00 (-0.01–0.01)	0.80	-0.00 (-0.01–0.00)	0.36	0.00 (-0.00–0.01)	0.18
Gender	0.09 -0.24–0.43)	0.59	0.06 (-0.26–0.39)	0.70	-0.04 -0.25–0.17)	0.73	0.02 (-0.30–0.34)	0.89
Education level	0.18*** (0.11–0.25)	<0.001	0.02 (-0.04–0.09)	0.50	0.02 (-0.03–0.06)	0.48	-0.04 (-0.10–0.03)	0.27
Income	0.02*** (0.01–0.03)	<0.001	0.02*** (0.01–0.03)	<0.001	-0.00 (-0.01–0.01)	0.75	-0.01 (-0.02–0.00)	0.10
Marital status	-0.10 (-0.34–0.14)	0.42	-0.01 (-0.25–0.22)	0.92	0.07 (-0.08–0.22)	0.38	-0.14 (-0.37–0.09)	0.24
Children	0.11 (-0.11–0.33)	0.33	-0.12 (-0.34–0.09)	0.27	0.10 (-0.04–0.24)	0.18	-0.11 (-0.32–0.10)	0.32
Cigarettes per day	-0.07 (-0.16–0.01)	0.09	0.00 (-0.08–0.09)	0.93	0.01 (-0.04–0.07)	0.62	0.11** (0.03–0.18)	0.01
Average waiting time from wakening to smoking the first cigarette	-0.01 (-0.08–0.06)	0.82	-0.04 (-0.11–0.03)	0.23	-0.04 (-0.09–0.01)	0.09	-0.07* (-0.14 – -0.00)	0.05
Adj R^2^	0.05		0.01		0.00		0.03	

Multiple regression models: all models account for demographic characteristics and nicotine dependence as control variables.

*** p≤0.001,

** p≤0.01,

* p≤0.05.

**Table 4:** The impact of rotating health warnings and reproductive health risk warning on general smoking risk perception, a survey experiment on smoking risk perception and quitting intentions, China, 2024 (N=1700)

	*Model 1* *(N=1121)* *(Group 1 vs 2)*		*Model 2* *(N=1138)* *(Group 1 vs 3)*	
	*Coefficient* *(95% CI)*	*p*	*Coefficient* *(95% CI)*	*p*
**Rotating health warnings / reproductive health risk warning**	0.28*** (0.12–0.43)	<0.001	0.09* (0.02–0.17)	0.02
**Control variables**				
Age (years)	0.00 (-0.01–0.01)	0.59	0.00 (-0.01–0.01)	0.85
Gender	-0.09 (-0.42–0.23)	0.57	-0.09 (-0.41–0.24)	0.61
Education level	-0.01 (-0.08–0.06)	0.76	-0.01 (-0.08–0.05)	0.70
Income	-0.00 (-0.01–0.01)	0.96	0.00 (-0.01–0.01)	0.97
Marital status	0.11 (-0.14–0.37)	0.38	0.02 (-0.22–0.25)	0.89
Children	-0.06 (-0.30–0.18)	0.64	0.08 (-0.13–0.30)	0.46
Cigarettes per day	-0.06 (-0.16–0.04)	0.22	-0.03 (-0.11–0.05)	0.46
Average waiting time from wakening to smoking the first cigarette	-0.04 (-0.11–0.03)	0.26	-0.00 (-0.07–0.07)	0.91
Adj R^2^	0.01		0.00	

Multiple regression models: all models account for demographic characteristics and nicotine dependence as control variables.

*** p≤0.001,

** p≤0.01,

* p≤0.05.

**Table 5:** The impact of rotating health warnings and reproductive health risk warning on general quitting intentions, a survey experiment on smoking risk perception and quitting intentions, China, 2024 (N=1700)

	*Model 3* *(N=1121)* *(Group 1 vs 2)*		*Model 4* *(N=1138)* *(Group 1 vs 3)*	
	*Coefficient* *(95% CI)*	*p*	*Coefficient* *(95% CI)*	*p*
**Specific messages**	0.55*** (0.41–0.70)	<0.001	0.27*** (0.20–0.34)	<0.001
**Control variables**				
Age (years)	0.01 (-0.00–0.01)	0.15	0.00 (-0.01–0.01)	0.73
Gender	-0.15 (-0.46–0.16)	0.34	-0.18 (-0.48–0.12)	0.24
Education level	0.00 (-0.07–0.07)	0.97	-0.02 (-0.08–0.04)	0.51
Income	0.00 (-0.01–0.01)	0.62	-0.00 (-0.01–0.01)	0.72
Marital status	0.03 (-0.21–0.27)	0.80	0.00 (-0.21–0.22)	0.99
Children	-0.08 (-0.31–0.15)	0.52	0.08 (-0.12–0.28)	0.43
Cigarettes per day	-0.06 (-0.15–0.04)	0.23	-0.07 (-0.14–0.01)	0.08
Average waiting time from wakening to smoking the first cigarette	-0.01 (-0.08–0.06)	0.71	-0.03 (-0.10–0.03)	0.29
Adj R^2^	0.05		0.04	

Multiple regression models: all models account for demographic characteristics and nicotine dependence as control variables.

*** p≤0.001,

** p≤0.01,

* p≤0.05.

Table 2 reports the estimated results for participants’ perceptions of smoking-related risks across different physiological systems. Following the rotating health risk text intervention, participants’ perceptions of cardiovascular system risks significantly increased (β_cardiovascular system_=0.20; 95% CI: 0.05–0.35, p≤0.01), as did their perceptions of digestive system risks (β_digestive_ system=0.22; 95% CI: 0.07–0.37, p≤0.01), respiratory system risks (β_respiratory system_=0.17; 95% CI: 0.07–0.26, p≤0.001), and reproductive system risks (β_reproductive_ system=0.21; 95% CI: 0.06–0.37, p≤0.01). This indicates that, compared to the control group, exposure to the rotating health risk text leads to a stronger perception of health risks.

Table 3 presents the estimated results for participants’ perceptions of smoking-related risks across different physiological systems. The findings indicate that following the addition of reproductive health risk text, participants’ perceptions of reproductive system risks significantly increase (β_reproductive system_=0.18; 95% CI: 0.10–0.25, p≤0.001), while their perceptions of risks associated with other systems remain unchanged.

Table 4 presents the estimated results for overall perceptions of smoking-related risks. Model 1 indicates that the rotating health risk text intervention has a significant impact on participants’ perceptions of smoking-related risks (β_model1_=0.28; 95% CI: 0.12–0.43, p≤0.001). In comparison, Model 2 shows that the addition of the reproductive health risk text also significantly influences smokers’ perceptions of smoking-related risks (β_model2_=0.09; 95% CI: 0.02–0.17, p≤0.05). However, the significance of the reproductive health risk text intervention is relatively weak. Across both models, no control variables demonstrated a significant influence on perceptions of smoking-related risks.

Table 5 presents the estimated results for respondents’ intentions to quit smoking. Model 3 shows that the rotating health risk text has a strongly significant effect on respondents’ intentions to quit smoking β_model3_=0.55; 95% CI: 0.41–0.70, p≤0.001). Similarly, Model 4 indicates that the addition of the reproductive health risk text significantly increases respondents’ intentions to quit smoking, also with strong significance (β_model4_=0.27; 95% CI: 0.20–0.34, p≤0.001). Across both models, no control variables were found to have a significant impact on intentions to quit smoking.

## DISCUSSION

This study demonstrates that both rotating health risk text and non-rotating specific health risk text significantly enhance smokers’ health risk perceptions and intentions to quit smoking, though they differ in terms of effectiveness and applicability. First, rotating health risk text comprehensively improves smokers’ perceptions of cardiovascular, digestive, respiratory, and reproductive system risks, showing its broad scope and comprehensive intervention effects. In contrast, the non-rotating reproductive health risk text focuses primarily on increasing perceptions of reproductive system risks, with no significant effects observed for other health risks. In terms of overall health risk perceptions, while both interventions are effective, the rotating text has a stronger significance, underscoring its superiority in information delivery and cognitive enhancement.

Regarding intentions to quit smoking, both rotating health risk text and non-rotating reproductive health risk text significantly increase smokers’ quitting intentions, with similarly high levels of statistical significance. Notably, both interventions demonstrate similarly high levels of statistical significance in their effect on quitting intentions, suggesting that when implementing rotating text proves challenging, non-rotating specific health risk text can serve as an effective alternative. This finding offers policymakers a practical and actionable option for tobacco control initiatives^22^.

A comparison with existing literature further validates the findings of this study. Hammond et al.^10^ highlighted that rotating health warning labels maintain the novelty of the information, sustaining smokers’ attention and broadening their awareness of multiple health risks. Similarly, Fathelrahman et al.^[Bibr CIT0023]23^ found in a multinational study that diverse warning messages are more likely to evoke emotional responses, prompting deeper reflection and enhancing health risk perceptions. The findings of this study align with these conclusions, affirming that implementing rotating health risk text effectively improves the comprehensiveness and persistence of health risk awareness.

However, for non-rotating reproductive health risk text, while its impact is limited to reproductive system risks, its effectiveness in increasing quitting intentions remains noteworthy. Zhang et al.^[Bibr CIT0005]5^ found that disseminating information about less recognized health risks, such as reproductive health hazards, can generate significant warning effects among younger populations^5^. This study extends this conclusion, confirming that reproductive health risk information also has significant effects among a broader age range of adult smokers. Furthermore, Yang et al.^[Bibr CIT0018]18^ pointed out that Chinese smokers’ awareness of reproductive health risks is substantially lower than that of lung cancer or cardiovascular disease. This gap in awareness provides an essential entry point for targeted interventions using specific health risk text.

It is also important to consider psychological reactance, a critical factor in the design of health warning messages. Previous research suggests that high-threat graphic warnings may provoke psychological resistance among smokers, thereby reducing intervention effectiveness^24-26^. Erceg-Hurn et al.^[Bibr CIT0027]27^ found that text-based warnings are less likely to trigger reactance compared to graphic warnings, making individuals more willing to accept and process the health information. This study adopts text-based health warnings, and the results demonstrate positive intervention effects for both text types, indirectly supporting the advantages of text warnings in minimizing psychological resistance. This outcome provides valuable practical guidance for the design of health warning messages.

From a policy and practical perspective, rotating health risk text, with its clear advantage in enhancing health risk perceptions, should be prioritized as a key direction for future tobacco health warning policies. However, given the high cost and practical difficulties associated with implementing rotating warnings, non-rotating specific health risk text – such as reproductive health risks – can serve as a feasible and cost-effective alternative. This strategy is particularly relevant in countries where tobacco control policies face economic or political challenges. By prioritizing the dissemination of information on less recognized health risks, policymakers can achieve greater marginal effects, ultimately advancing the effective implementation of tobacco health warning policies.

### Limitations

This study has several limitations. First, the crosssectional design limits the ability to evaluate the long-term effects of health warning texts. It remains unclear whether these interventions will sustain their influence on smokers’ risk perceptions and quitting behaviors over time. Second, this study simplifies the implementation of rotating health warnings by simultaneously presenting multiple warning texts in a questionnaire, rather than simulating real-world market conditions where health warnings rotate periodically. This simplification may impact the external validity of our findings, as it does not fully replicate the dynamic exposure to warnings experienced in real-world settings.

Another limitation is the potential for residual confounding. Despite controlling for several known factors, unmeasured or unknown variables may still influence the outcomes, potentially affecting the robustness of the findings. Additionally, the generalizability of the results is limited, as the sample was drawn from a specific population within a single country. Therefore, the findings may not be directly applicable to other countries or cultural contexts with different health communication practices or smoking behaviors. Furthermore, the study used snowball sampling to recruit participants, which may introduce selection bias, limiting the external validity of the findings.

Finally, while multiple linear regression models were employed to analyze the data, it is important to note that the use of this approach with ordinal variables (e.g. smokers’ intentions or risk perceptions) may not always be appropriate. Future studies could consider using statistical techniques specifically designed for ordinal data, such as ordinal logistic regression, to better account for the nature of these variables and provide more accurate results.

## CONCLUSIONS

This study demonstrates that both rotating health risk text and non-rotating specific reproductive health risk text significantly enhance smokers’ perceptions of smoking-related risks and their intentions to quit smoking. The rotating health risk text intervention significantly increases perceptions of cardiovascular, digestive, respiratory, and reproductive system risks, while the non-rotating reproductive health risk text only improves perceptions of reproductive system risks, with no significant effect on other risk perceptions. In terms of overall smoking-related health risk perceptions, both interventions are effective, but the rotating text shows a stronger significance. Regarding intentions to quit smoking, both interventions significantly increase smokers’ quitting intentions, with similarly high levels of significance. Notably, both interventions demonstrate similarly high levels of statistical significance in their effect on quitting intentions, indicating comparable effectiveness in this regard.

## Data Availability

The data supporting this research are available from the authors on reasonable request.
